# Effect of Clinical Typing on Serum Urate Targets of Benzbromarone in Chinese Gout Patients: A Prospective Cohort Study

**DOI:** 10.3389/fmed.2021.806710

**Published:** 2022-01-17

**Authors:** Xiaomei Xue, Xuan Yuan, Lin Han, Xinde Li, Tony R. Merriman, Lingling Cui, Zhen Liu, Wenyan Sun, Can Wang, Fei Yan, Yuwei He, Aichang Ji, Jie Lu, Changgui Li

**Affiliations:** ^1^Department of Endocrinology and Metabolism, The Affiliated Hospital of Qingdao University, Qingdao, China; ^2^Institute of Metabolic Diseases, Qingdao University, Qingdao, China; ^3^Shandong Provincial Key Laboratory of Metabolic Diseases and Qingdao Key Laboratory of Gout, The Affiliated Hospital of Qingdao University, Qingdao, China; ^4^Department of Biochemistry, University of Otago, Dunedin, New Zealand; ^5^Division of Clinical Immunology and Rheumatology, University of Alabama Birmingham, Birmingham, AL, United States; ^6^Shandong Provincial Clinical Research Center for Immune Diseases and Gout, Qingdao, China

**Keywords:** gout, clinical typing, serum urate target, low-dose benzbromarone, urate-lowering therapy

## Abstract

**Introduction:**

Achieving a goal of serum urate levels in patients with gout is an important way to prevent gout and its complications while it remains difficult with a low targeting rate worldwidely. Currently, hyperuricemia classification has not been widely applied to the management of gout owing to insufficient clinical evidences. This study aimed to evaluate the effectiveness of achieving target urate based on hyperuricemia classification in Chinese patients with gout.

**Methods:**

In this prospective study, patients with gout receiving urate lowering therapy with benzbromarone were assigned to two groups, a renal underexcretion and an unclassified type. The primary endpoint was the proportion of patients achieving the serum urate target (<360 μmol/L) during the 12-week study. The frequency of acute gout attacks as well as physical and chemical indicators were secondary endpoints.

**Results:**

Target serum urate level was achieved in 60.5% of underexcretors compared with 39.0% of patients of the unclassified type at week 12 (*P* = 0.002). Blood glucose and cholesterol levels were lower in the underexcretor group compared with the unclassified type group at the end of the trial, without significant different frequencies in gout flare during the study. In subgroup analysis, stratified by body mass index and estimated glomerular filtration rate, the proportion of patients with serum urate <360 μmol/L was greater in the underexcretion compared with the unclassified type group.

**Conclusions:**

The increased achievement of target serum urate in the underexcretion group supports the use of a clinical hyperuricemia typing treatment strategy for gout.

## Introduction

Gout is a disease with a metabolic basis primarily caused by reduced urate excretion (1). Gout leads to joint deformity, and is associated with kidney damage (2) (uremia), and induces or aggravates diabetes (3) and cardiovascular diseases (4) when hyperuricemia in patients with gout is inadequately treated. The goal of gout therapy is to lower serum urate (SU) below the threshold of supersaturation (<360 μmol/L) to prevent any gouty attack by allowing the dissolution of existing monosodium urate (MSU) crystals (5), as well as to improve the heart and kidney complications (6, 7). However, the poor adherence to urate-lowering therapy (ULT) and clinical inertia induces a poor achievement of target SU (8–13), which puts forward new challenges to clinical diagnosis and treatment.

Hyperuricemia can be classified into the underexcretion type, the overproduction type, the combined type, and the extra-renal urate underexcretion type according to the fractional excretion of urate (FEUA) and 24-h urinary urate excretion (UUE) (14–16). Previous studies have indicated that the drug selection by hyperuricemia classification type can improve reduction in SU levels (17–19). But these findings have not been confirmed in an independent clinical trial.

Benzbromarone is a first-line urate-lowering drug in Asia, especially in China, while a second-line drug in European and American countries (20, 21). Given the large population treated with benzbromarone, it is particularly crucial to take hyperuricemia classification, for benzbromarone is not indicated for the patients caused by uric acid overproduction (22).

Thus far, only the 2006 European League Against Rheumatism (EULAR) evidence-based recommendations for gout suggest renal urate excretion should be determined to inform treatment in selected patients with gout, especially those with a family history of young onset gout, onset of gout under age 25, or with renal calculi (level IIb evidence) (23), while the 2020 American College of Rheumatology (ACR) Gout Clinical Practice Guidelines and 2020 recommendations from the French Society of Rheumatology for the management of gout advise against checking urine urate level for patients with gout due to the lack of evidence (24, 25).

Currently, the lack of clinical evidence hinders the application of hyperuricemia classification to the treatment of gout. The present study was designed to evaluate the effectiveness of treatment based on urate excretion classification in Chinese patients with gout with low-dose benzbromarone.

## Methods

### Study Design and Patients

This single-center, controlled prospective clinical study was conducted in the dedicated Shandong Gout Clinic Medical Center at the Affiliated Hospital of Qingdao University. The clinical trial was conducted according to the principles from the Declaration of Helsinki, and approved by the Ethics Committee of the institution and registered in ChiCTR (#1900022981). Two hundred and forty Chinese male patients with gout were recruited between May 2019 and July 2020. All patients who participated in this study provided written informed consent. We enrolled patients who met the 2015 ACR/EULAR diagnostic criteria of primary gout (26) with 18–70 years of age. As the female patients were limited in our clinic and to minimize the influence of sex confounding, we only included males in this study. Key exclusion criteria were (1) SU <420 μmol/L or SU > 600 μmol/L, (2) estimated glomerular filtration rate (eGFR) <60 mL/min/1.73 m^2^, (3) presenting abnormally high levels of transaminases (> 2 times of the upper limit of normal, ULN), (4) suffering from renal calculus, polycystic kidney, rheumatoid arthritis, diabetes, or other serious complications, and (5) use of drugs affecting SU levels, such as losartan, fenofibrate, metformin, and hydrochlorothiazide. The reasons to exclude patients with gout with SU > 600 μmol/L are considerations of heavy burden to kidney with uricosuric agents and decreased efficacy of low-dose benzbromarone in the management of such high SU levels (27).

### Assessments and Procedures

Before the start of the trial, all the participants were required to undertake a 2-week washout. During the period, the patients maintained a low purine diet and took no drugs. If there was a gout flare within the washout duration, the patients were prescribed colchicine or etoricoxib and restarted the washout process. Participants who underwent the classification test were required to conduct a 24-h urine collection 1 day prior to baseline visit. The total urinary volume in 24 h was also recorded.

Basic characteristics, including age, height, weight, disease duration of gout, frequency of attacks (per year), coexisting conditions, smoking history, alcohol drinking history, were collected. SU, blood urea nitrogen (BUN), serum creatinine (sCr), serum glucose (GLU), serum triglyceride (TG), serum cholesterol (TC), serum alanine aminotransferase (ALT), serum aspartate aminotransferase (AST), 24-h urine urate (uUA), and creatinine (uCr) were measured using an automatic biochemical analyzer (TBA-40FR, Toshiba Company, Japan). Body mass index (BMI) was calculated as weight in kilograms divided by height in meters squared. eGFR was calculated using the Modification of Diet in Renal Disease formula (28). The FEUA and 24-h UUE were calculated by 24-h urine volume and 24-h uUA and uCr. FEUA= uUA/uCr × sCr/sUA × 100 % and UUE = uUA × 24-h urinary volume / [0.0061 × height (cm) + 0.0128 × weight (kg) – 0.1529) × 1.73 (mg/d/1.73m^2^)] (29). The underexcretion type of hyperuricemia was defined as the FEUA <5.5% and 24-h UUE ≤ 600 mg/day/1.73 m^2^ (16, 29).

The same regime was implemented in both groups, i.e., 25 mg benzbromarone qd. and 1 g sodium bicarbonate tid. for 12 weeks. The patients were encouraged to drink water ≥ 2,000 mL daily. Follow-ups and biochemical measurements were requested at weeks 2, 4, 8, and 12 (Supplementary Figure 1). All patients were given low dose of colchicine (0.5 mg/day) during the trial to prevent acute attacks of gout. The patients were withdrawn if there was evidence of severe adverse effects in the liver or kidney, including increased hepatic enzymes to three times the normal range, eGFR ≤ 60 mL/min/1.73 m^2^, and any symptoms that indicated poor tolerance to treatment.

### Outcomes

The main outcomes were the SU level in each follow-up time point and the proportion of patients achieving the SU target (SU <360 μmol/L). The frequency of acute gout attack, physical, and chemical indicators (blood and urine routine, liver and kidney function, etc.) were the secondary endpoints. Associations of final SU and clinical variables were analyzed. Safety data were recorded at every follow-up time.

### Data Analysis

A total of 117 patients were required for each group to detect a difference in the proportion of patients with SU <360 μmol/L with a 5% two-sided significance and 90% power based on data from the previous (20). This allows a 10% dropout rate over the period of the study. Since approximately two thirds of patients with gout were found as underexcretors in studies (30), 175 were collected and 120 were allocated to the underexcretion group.

All data were analyzed using the Statistical Product and Service Solutions v22.0 (IBM SPSS, Chicago, USA) software. Clinical characteristics were summarized using standard descriptive statistics including mean (SD), median (interquartile range, IQR), and number (proportion). We employed a mixed model to analyze the variation trend of the two groups with repeated measurement data. The computing methods of this mixed model emphasized the comparison of the changing trend of variables, eGFR, sCr, BUN, TG, and other indicators, between the two groups over the main effect of time. Stepwise linear regression analysis was used to determine the independent predictors associated with SU levels at the final study visit with an inclusion of individual components (urate excretion determination informed, positive gout family history, age, onset age, baseline SU, BMI, and eGFR). Data from regression analyses were summarized using standardized β coefficients (95% CI). *P* < 0.05 was considered statistically significant.

## Results

### Patients' Characteristics

**Figure 1:**
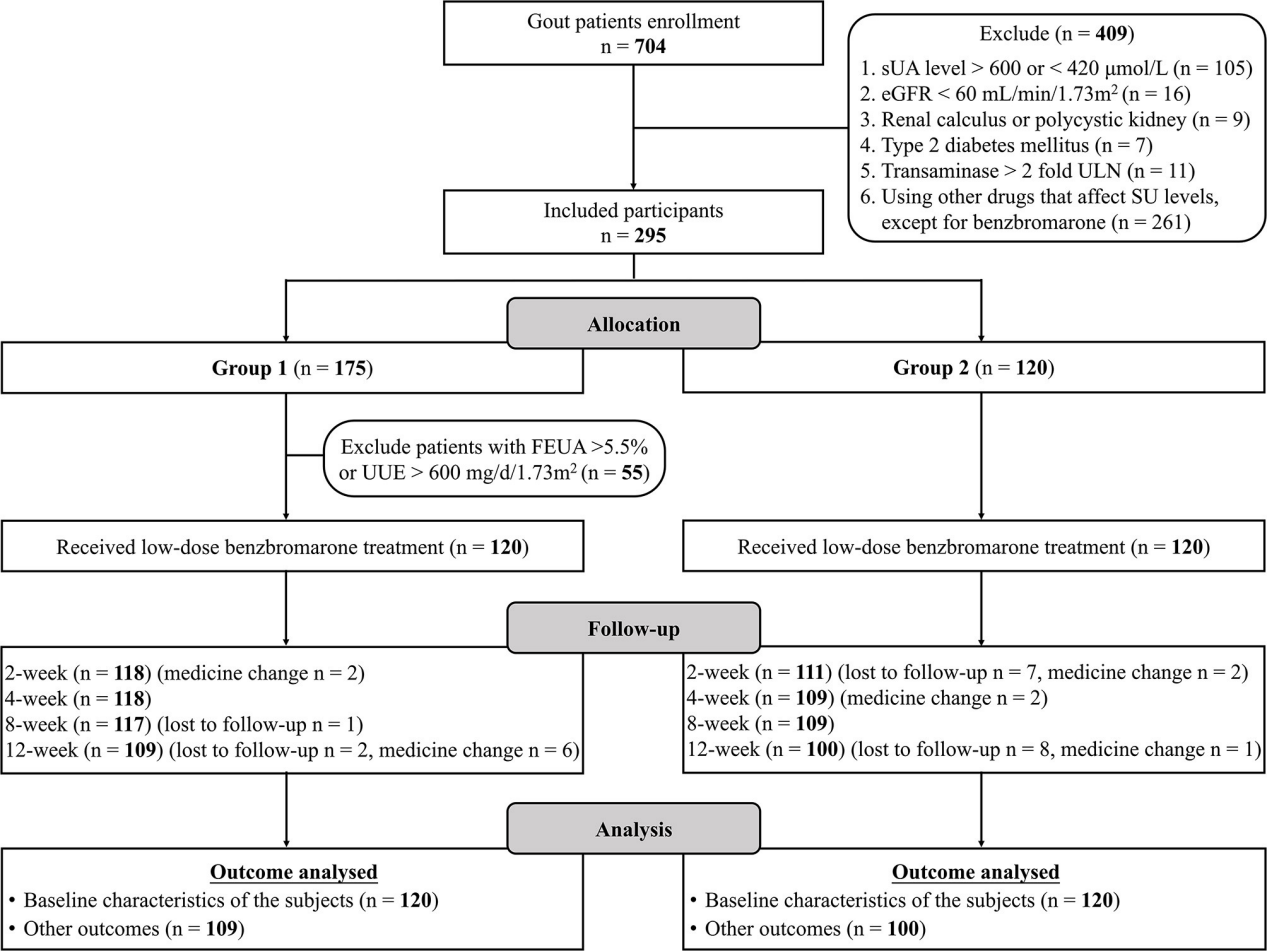
Study flow chart. SU, Serum urate; eGFR, estimated glomerular filtration rate; SU, serum urate; FEUA, fractional excretion of urate; UUE, urinary urate excretion.

There were 704 patients referred for consideration for entry into the study (Figure 1). Reasons for exclusion were: SU <420 μmol/L or SU > 600 μmol/L (*n* = 105), eGFR <60 mL/min/1.73m^2^ (*n* = 16), transaminase > 2-fold of the ULN (*n* = 11), renal calculus or polycystic kidneys (*n* = 9), type 2 diabetes mellitus (*n* = 7), and prescribed drugs affecting SU levels (*n* = 261). We recruited 175 patients for the underexcretion type group and 120 matched patients for the unclassified type group. Clinical typing filtered out 120 underexcretors from 175 patients as 55 with FEUA ≥ 5.5% and/or 24-h UUE > 600 mg/day/1.73m^2^. A total of 31 participants were lost to follow-up or adjusted their medication regime. Ultimately, 209 patients (unclassified type group: *n* = 100, underexcretion type group: *n* = 109) completed 12 weeks of ULT and were included in the analysis (Figure 1). Baseline characteristics of participants were comparable between the two groups (Table 1).

**Table 1:** Baseline characteristics of the subjects.

	**Unclassified** **type** **(*n* = 120)**	**Underexcretion** **type** **(*n* = 120)**
Demographic and general characteristics		
Age (years), mean (SD)	46.13 (13.51)	44.88 (12.74)
Gout onset age (years), mean (SD)	40.85 (12.22)	39.65 (11.51)
BMI (kg/m^2^), mean (SD)	26.16 (2.89)	26.13 (2.87)
Frequency of attacks (per year), median (IQR)	1 (1, 2.5)	1 (1, 2.75)
Coexisting conditions		
Hypertension, n (%)	23 (19.20)	22 (18.30)
Cardiovascular disease, n (%)	8 (6.70)	3 (2.50)
Fatty liver, n (%)	32 (26.70)	21 (17.50)
Hyperlipidemia, n (%)	42 (35.00)	32 (26.70)
Tophus, n (%)	12 (10)	17 (14.20)
Family history of gout, n (%)	30 (26.80)	24 (20.00)
Lifestyles		
Smoking history, n (%)	27 (22.50)	32 (26.70)
Alcohol history, n (%)	47 (39.20)	59 (49.20)
Blood chemistry parameters		
Serum urate (μmol/L), mean (SD)	506.92 (48.60)	499.35 (47.80)
eGFR (mL/min/1.73 m^2^), mean (SD)	98.63 (17.03)	98.37 (18.19)
Serum creatinine (μmol/L), mean (SD)	80.43 (10.62)	81.21 (11.45)
Blood urea nitrogen (mmol/L), mean (SD)	4.61 (1.09)	4.18 (0.89)
Triglyceride (mmol/L), mean (SD)	1.84 (0.86)	1.81 (0.84)
Cholesterol (mmol/L), mean (SD)	5.02 (0.97)	4.82 (0.83)
Fasting Glucose (mmol/L), mean (SD)	5.47 (0.54)	5.40 (0.52)
ALT (U/L), median (IQR)	22 (17.25, 33.75)	24 (19, 33)
AST (U/L), median (IQR)	20 (18, 24)	19 (17, 22)

*Data were presented as the mean (±SD) or interquartile range.*

*BMI, body mass index; eGFR, estimated glomerular filtration rate; ALT, alanine aminotransferase; AST, aspartate aminotransferase; IQR, interquartile range*.

### Primary Outcomes

**Figure 2:**
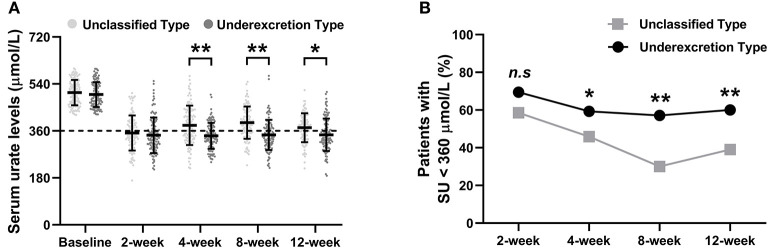
The primary outcomes of the urate-lowering therapy based on hyperuricemia classification. **(A)** The trend of serum urate level and **(B)** proportion of patients with SU < 360 μmol/L during the 12-week study. SU levels are presented as the mean (±SD). Rates are expressed as proportions. SU, serum urate. Symbols * and ** indicate *P* < 0.05 and < 0.01 between the unclassified and underexcretion groups, respectively.

Obvious SU reductions were observed after the first 2 weeks of ULT in both the unclassified type group and the underexcretion type group (Figure 2A). Further reductions in SU levels were observed at the 4-, 8-, and 12-week time points (Figure 2A). The mean (± SD) final SUs were 372.24 (± 55.50) μmol/L in the unclassified type group and 349.73 (± 62.96) μmol/L in the underexcretion group (each *P* < 0.001 compared with baseline) (Figure 2A). Compared with the unclassified type group, the underexcretion type group showed significantly lower levels in SU after 4, 8, and 12 weeks (*P* < 0.001, *P* < 0.001, and *P* = 0.01, respectively) (Figure 2A).

The proportion of patients who achieved the SU target (SU <360 μmol/L) increased dramatically after 2 weeks of ULT and remained at this level at the 4-, 8-, and 12-week time points (Figure 2B). Significant differences in the percentage of patients who achieved the SU target between the underexcretion type group and the unclassified type group were observed at the 4-week (59.3% *vs*. 45.9%, *P* = 0.04), 8-week (57.1% *vs*. 30.0%, *P* < 0.001), and 12-week time points (60.5% *vs*. 39.0%, *P* = 0.002) (Figure 2B).

### Secondary Outcomes

No significant changes in the frequency of gout flare, eGFR, sCr, TG, fasting GLU, ALT, or AST were observed during the trial (Table 2). However, the levels of BUN increased from 4.65 (± 1.11) mmol/L at baseline to 4.98 (± 1.03) mmol/L at the 12-week time point in the unclassified type group (*P* = 0.004) (Table 2). When compared with the unclassified type group, the underexcretion type group had lower levels of TG, TC, and fasting GLU (Table 2). Specifically, mean TG in the underexcretion type group was 0.22 mmol/L lower than in the unclassified type group at the 8-week time point (*P* = 0.03) (Table 2). Mean TC in the underexcretion type group was 0.31 mmol/L lower than in the unclassified type group at the 8-week time point and 0.30 mmol/L lower at the 12-week time point (both *P* = 0.01) (Table 2). Mean fasting GLU levels showed a similar decrease in the underexcretion type group compared with the unclassified type group from the 4th week on and continued until the 12th week (*P* = 0.01, *P* < 0.001, and *P* = 0.002, respectively) (Table 2).

**Table 2:** Changes in the clinical characteristics during the trial.

	**Baseline**	**4 weeks**	**8 weeks**	**12 weeks**	***P* trend**
Gout flare, n (%)					
Unclassified type	-	11 (9.2)	8 (6.7)	9 (7.5)	0.49
Underexcretion type	-	16 (13.3)	10 (8.3)	13 (10.8)	0.53
*P* value		0.31	0.62	0.37	
eGFR (mL/min/1.73 m^2^), mean (SD)					
Unclassified type	98.63 (17.03)	98.50 (16.11)	97.71 (16.25)	97.26 (15.87)	0.88
Underexcretion type	98.37 (18.19)	98.28 (19.57)	98.02 (18.61)	97.57 (22.98)	0.37
*P* value	0.81	0.37	0.89	0.91	
Serum creatinine (μmol/L), mean (SD)					
Unclassified type	80.43 (10.62)	80.33 (10.09)	81.02 (10.44)	81.15 (10.18)	0.82
Underexcretion type	81.21 (11.45)	81.03 (13.90)	81.53 (11.47)	82.35 (12.38)	0.79
*P* value	0.58	0.66	0.72	0.43	
Blood urea nitrogen (mmol/L), mean (SD)					
Unclassified type	4.65 (1.11)	4.64 (1.13)	4.85 (1.22)	4.98 (1.03)	0.004
Underexcretion type	4.18 (0.89)	4.75 (1.26)	4.74 (1.21)	4.89 (1.28)	0.11
*P* value	0.002	0.50	0.49	0.42	
Triglyceride (mmol/L), mean (SD)					
Unclassified type	1.83 (0.79)	1.90 (0.95)	1.94(1.00)	1.95 (0.89)	0.37
Underexcretion type	1.82 (0.84)	1.78 (0.96)	1.72 (0.92)	1.89(1.41)	0.29
*P* value	0.63	0.28	0.04	0.83	
Cholesterol (mmol/L), mean (SD)					
Unclassified type	5.02 (0.97)	5.04 (0.91)	5.16 (0.96)	5.14 (0.95)	0.05
Underexcretion type	4.82 (0.83)	4.85 (0.89)	4.85 (0.87)	4.84 (0.85)	0.97
*P* value	0.09	0.10	0.01	0.01	
Fasting Glucose (mmol/L), mean (SD)					
Unclassified type	5.47 (0.54)	5.55 (0.47)	5.60 (0.47)	5.57 (0.40)	0.28
Underexcretion type	5.40 (0.52)	5.36 (0.66)	5.33 (0.49)	5.39 (0.49)	0.44
*P* value	0.19	0.01	<0.001	0.002	
ALT (U/L), median (IQR)					
Unclassified type	22 (17, 34)	23 (17, 33)	23 (17, 33)	22 (17, 30)	0.44
Underexcretion type	24 (19, 33)	23 (18, 33)	24 (17, 33)	23 (17, 31)	0.30
*P* value	0.50	0.63	0.80	0.76	
AST (U/L), median (IQR)					
Unclassified type	20 (18, 24)	20 (16, 24)	20 (17, 24)	20 (17, 25)	0.40
Underexcretion type	19 (17, 22)	19 (17, 24)	20 (16, 23)	20 (17, 23)	0.13
*P* value	0.05	0.33	0.64	0.30	

*Data were presented as the mean (±SD), interquartile range or numbers (percentage).*

*BMI, body mass index; eGFR, estimated glomerular filtration rate; ALT, alanine aminotransferase; AST, aspartate aminotransferase; IQR, interquartile range.*

*P trend value was calculated using a mixed ANOVA model*.

### Association of Final SU With Clinical Variables in Linear Regression Analysis

During the 12-week follow-up study, no participants were taking drugs (other than benzbromarone) that could influence SU levels. In the entire group, linear regression analysis showed that the factors associated with changes in SU levels at the final study visit were urate excretion determination typing (standardized β: −0.15, 95% CI: −0.29 ~ −0.02, *P* = 0.03), baseline SU (standardized β: 0.17, 95% CI: 0.03 ~ 0.31, *P* = 0.02), and BMI (standardized β: 0.20, 95% CI: 0.07 ~ 0.36, *P* = 0.004) (Table 3). For participants in the unclassified type group, only BMI (standardized β: 0.24, 95% CI: 0.03 ~ 0.50, *P* = 0.03) was an independent predictor of SU, accounting for 5.8% of SU variance (Table 3), while the independent predictors in the underexcretion type group included eGFR (standardized β: −0.19, 95% CI: −0.38 ~ −0.01, *P* = 0.04) and BMI (standardized β: 0.22, 95% CI: 0.05 ~ 0.42, P = 0.02), accounting for 7.8% of SU variance (Table 3).

**Table 3:** Predictors of serum urate on the 12-week in linear regression analysis.

	**Variable**	**Standardized β**	**95% CI**	***P***	**Model summary**
**All patients**	Urate excretion determination (yes)	−0.15	−0.29~-0.02	0.03	*R*^2^ = 0.10, *F* = 7.23, *P* <0.001
	BMI	0.20	0.07~0.36	0.004	
	Baseline SU	0.17	0.03~0.31	0.02	
**Unclassified type**	BMI	0.24	0.03~0.50	0.03	*R*^2^ = 0.06, *F* = 5.21, *P* = 0.03
**Underexcretion type**	BMI	0.22	0.05–0.42	0.02	*R*^2^ = 0.08, *F* = 4.83, *P* = 0.01
	eGFR	−0.19	−0.38~-0.01	0.04	

*The following variables were included in the model: baseline SU (60 μmol/L for a unit), urate excretion determination (yes/no), gout family history (yes/no), age, onset age, body mass index, and estimated glomerular filtration rate.*

*BMI, body mass index; eGFR, estimated glomerular filtration rate*.

### Subgroup Analysis Based on BMI and eGFR

Baseline SU levels, BMI, and urate excretion determination typing were suggested to be predictors, independent of other covariates, of SU at week 12 in the combined cohort, while BMI in the unclassified type group, and baseline BMI and eGFR in the underexcretion type group provided evidence as independent predictors (Table 3). We then performed a subgroup analysis based on the BMI (> or ≤ 25 kg/m^2^) and eGFR (> or ≤ 90 mL/min/1.73 m^2^).

**Figure 3:**
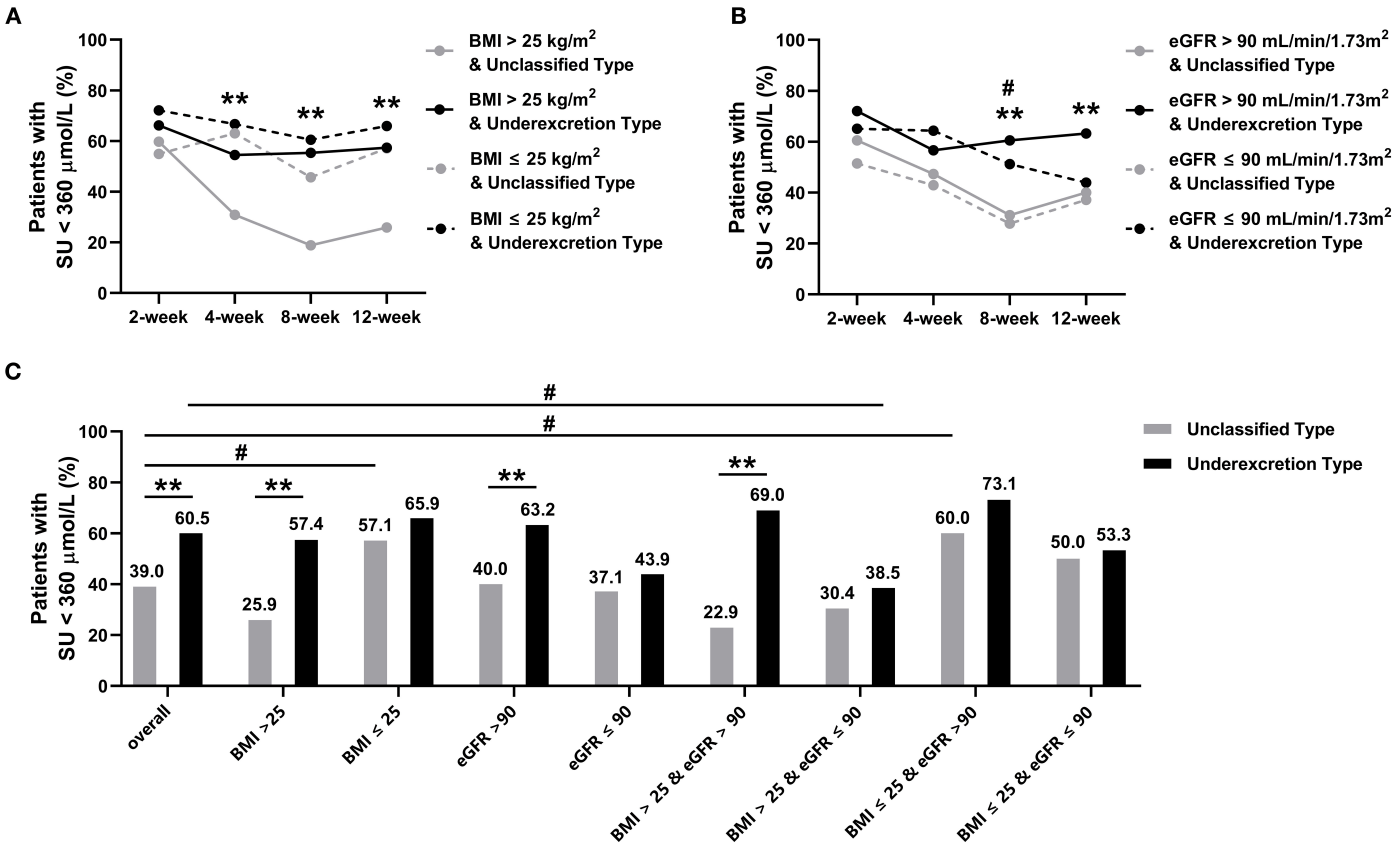
Proportion of patients with serum urate (SU) < 360 μmol/L in subgroups during the 12-week study. **(A)** Subgrouped by BMI (>25 kg/m^2^ or ≤ 25 kg/m^2^), symbols ^*^ and ^**^ indicate *P* < 0.05 and < 0.01 in BMI > 25 kg/m^2^ between unclassified and underexcretion groups, respectively. **(B)** Subgrouped by eGFR (> 90 mL/min/1.73 m^2^ or ≤ 90 mL/min/1.73 m^2^), symbols ^*^ and ^**^ indicate *P* < 0.05 and < 0.01 in eGFR > 90 ml/min/1.73 m^2^ between unclassified and underexcretion groups, respectively; symbols ^#^ and ^##^ indicate *P* < 0.05 and < 0.01 between eGFR ≤ 90 ml/min/1.73 m^2^ in unclassified and underexcretion groups, respectively. **(C)** Subgrouped by BMI and eGFR (BMI > 25 kg/m^2^ and eGFR > 90 mL/min/1.73 m^2^, BMI > 25 kg/m^2^ and eGFR ≤ 90 mL/min/1.73 m^2^, BMI ≤ 25 kg/m^2^ and eGFR > 90 mL/min/1.73 m^2^, BMI ≤ 25 kg/m^2^ and eGFR ≤ 90 mL/min/1.73 m^2^), symbols ^*^ and ^**^ indicate *P* < 0.05 and < 0.01 between unclassified and underexcretion groups within same subgroups, respectively, symbol ^#^ indicates *P* < 0.05 between overall and subgroups within unclassified or underexcretion groups. eGFR, estimated glomerular filtration rate; BMI, body mass index; SU, serum urate.

Stratified analysis results revealed a higher proportion of patients with SU <360 μmol/L in the underexcretion type group than in the unclassified type group, irrespective of the BMI and eGFR grouping, which was manifested by the subgroup of patients with BMI > 25 kg/m^2^ and eGFR > 90 mL/min/1.73 m^2^ (Figure 3). The proportion was significantly higher in the patients with BMI ≤ 25 kg/m^2^ than those with BMI > 25 kg/m^2^ in the unclassified type group (57.1% *vs*. 25.9%, *P* = 0.002) (Figure 3A). In the underexcretion type group, the proportion was higher in the patients with eGFR > 90 mL/min/1.73 m^2^ than those with eGFR ≤ 90 mL/min/1.73 m^2^ (63.2% *vs*. 43.9%, *P* = 0.045) (Figure 3B). The highest proportion of patients in the underexcretion type achieving target SU was observed in patients with eGFR > 90 mL/min/1.73 m^2^ and BMI ≤ 25 kg/m^2^ (73.1%, Figure 3C). A significant gap of 46.1% of the proportion between the underexcretion type and the unclassified type was shown in the subgroup of eGFR > 90 mL/min/1.73 m^2^ and BMI > 25 kg/m^2^ (Figure 3C).

### Safety Profile

Safety assessments throughout the study included the incidence and severity of treatment-emergent adverse events (AEs). The proportions of AEs in the two groups were similar while no clinically important or serious AEs were reported (Supplementary Table 1).

During the 12-week trial, twenty-five (22.9%) patients had acute gout flares in the unclassified type group and 30 (30.0%) in the underexcretion type group (*P* = 0.25) (Supplementary Table 1). In the unclassified type group, 22 (20.2%) patients had one gout attack and 3 (2.8%) had two, while in the underexcretion type group, 22 (22.0%) had one flare, 7 (7.0%) had two, and 1 (1.0%) had more than two (Supplementary Table 1).

We measured the serum transaminase levels to assess hepatic safety. No significant changes in transaminase levels were observed over this period (Supplementary Table 1). Twenty-one (19.3%) in the unclassified type group and 17 (17.0%) patients in the underexcretion type group had elevated transaminase levels (*P* = 0.67) (Supplementary Table 1). During the trial, only five patients' serum transaminase levels were > 2 times upper limit of normal of transaminase, so they were given liver protection and only one withdrew because of > 3 times upper limit of normal of transaminase (Supplementary Table 1). After 1 week, the transaminase levels returned to the normal range, which indicated a well tolerance to low-dose benzbromarone.

Assessments of renal safety consisted of eGFR values and renal calculus. Only one patient experienced at least one renal-related AE in the 12-week study, showing eGFR <60 mL/min/1.73 m^2^ (Supplementary Table 1). New onset renal calculus was detected using urinary system ultrasound at the end of the trial. Eight (7.3%) patients in the unclassified type group and 6 (6.0%) in the underexcretion type group were reported with renal calculus (*P* = 0.70, Supplementary Table 1).

Altogether, three patients were recorded with gastrointestinal disorders, two with increased heart rate compared with before enrollment (80–100/min), one with itchy skin, and one with respiratory, thoracic, and mediastinal disorders in the underexcretion type group (Supplementary Table 1).

## Discussions

To the best of our knowledge, this is the first prospective cohort study to observe the impact of hyperuricemia classification-informed treatment on SU targets in Chinese primary patients with gout. In this clinical trial, we compared the effectiveness of target SU achievement as well as the safety of low-dose benzbromarone between a group of patients of the unclassified excretion type and those classified as underexcretion type for 12 weeks. Notably, the target SU was achieved by 60.5% of underexcretors compared with 39.0% of patients of the unclassified excretion type group at the 12th week (*P* = 0.002) without significant clinically important or serious AEs. Our data support the classification of HU in the treatment of gout, with improved proportions of achieving target SU.

Currently, the rate of successfully achieving the target SU level remains suboptimum, showing 37.5% in a Japanese cross-sectional study (31) and 35.7% in our previous clinical trial in which patients were treated with low-dose benzbromarone (20). Besides, a meta-analysis involving 137,699 patients with gout revealed that the overall adherence rate was only 47% (95% CI: 42%~52%, I^2^ = 99.7%) (32). These data may reflect poor compliance or non etiological target treatment. The worth of classification of hyperuricemia/gout has not been widely recognized although the 2006 EULAR gout guidelines and 2018 management consensus on hyperuricemia/gout proposed by Taiwan experts acknowledged the necessity of renal urate excretion determination (22, 23). Previous studies have indicated that for underexcretors, patients taking benzbromarone 100 mg/day showed a 58.27% decrease of percentage from initial SU compared with only 36.26% in patients taking allopurinol 300 mg/day (17). Another study also confirmed that low dose (40 mg) febuxostat was more effective in patients with urate overproduction than in underexcretors (19).

So far, renal urate underexcretion has been widely considered to be a main cause of hyperuricemia (30). Although the unclassified type group might contain a majority of underexcretors, the differences in SU decrease and proportion of SU <360 μmol/L were significant between the groups in our study. The increased proportions of patients achieving SU target in the underexcretion type group at each follow-up time point indicated a high effectiveness of low-dose benzbromarone for these patients. Our data showed a 1.5-fold increase in the proportion of patients achieving target SU, further confirming the advantages of hyperuricemia classification typed drug selection and showing an evidence-based suggestion in clinic.

In addition, our study also found that patients in the underexcretion type group had significantly lower blood glucose and cholesterol after 8-week ULT and afterward compared with those in the unclassified type group. This result can be explained by the fact that fasting glucose was positively correlated to SU when it is below the threshold (6.5 mmol/L in men and 7.5 mmol/L in women) (33). In addition, the indirect mechanism of insulin resistance may also be involved in the results (34, 35).

Association by linear regression analysis of all enrollments showed that patients in the underexcretion type group were more likely to reach the target of SU than those in the unclassified type group. Our study found that baseline SU level was a predictor independent of included covariates for the final SU level in all participants. This is consistent with our previous research (20). In addition, the final SU level in the unclassified type group was also associated with BMI at baseline, while in the underexcretion type group was also associated with eGFR at baseline. It has been reported that BMI is a risk factor for hyperuricemia independent of covariates (36). Moreover, Yamashita et al. (37) showed an association between BMI and renal handling of urate, suggesting that hyperuricemia in obese people is mainly attributed to an impaired renal clearance of urate rather than overproduction. An association between a high BMI and a lower FEUA has been evidenced in Eastern Polynesian and New Zealand European participants as well (15). The stratified analysis of eGFR showed that the proportion of achieving SU target in patients with eGFR > 90 mL/min/1.73 m^2^ was significantly higher than that of patients with eGFR ≤ 90 mL/min/1.73 m^2^ in the underexcretion type group. This can be explained by the fact that a strong renal function indicates a good renal handling of urate (38).

The latest ACR gout diagnosis and treatment guidelines further emphasize the importance of reaching the target of SU and the necessity of taking lifelong urate-lowering medication. Therefore, from the consideration of long-term maintenance of target SU, there is an ongoing need to lower the level of SU more effectively. We strongly recommend that the classification for type-leading treatment is etiologically consistent and abides by the principle of precision therapy. The urate-lowering efficacy was comparable between 25 mg benzbromarone qd. and 20 mg febuxostat qd. (20). Fujimori et al. (39) suggest that benzbromarone is applicable to the management of hyperuricemia associated with renal impairment even with long-term use.

We acknowledge the limitations of this study. Our study was a single-center clinical trial and should be confirmed in multiple centers. The results may not be generalizable to other ethnic groups and to people living in other countries. Currently, we only compared the efficacy of benzbromarone administration, in the absence of a comparison with allopurinol or febuxostat.

## Conclusions

In summary, this study has demonstrated the positive effect of clinical classification of gout on SU targets. High effectiveness without significant treatment-emergent AEs was concluded in the urate underexcretion group when using low-dose benzbromarone as the urate-lowering regime. These data provide further support for classification application in the type-leading treatment of patients with gout and provide evidence for the amendments of guidelines. Therefore, we propose that patients with gout need to be tested for clinical classification when choosing urate-lowering drugs. It was recommended that low-dose benzbromarone should be the first-line drug in urate underexcretors without renal insufficiency or other contraindications.

## Author's Note

All named authors meet the International Committee of Medical Journal Editors (ICMJE) criteria for authorship for this article, take responsibility for the integrity of the work as a whole, and have given their approval for this version to be published.

## Data Availability Statement

The original contributions presented in the study are included in the article/Supplementary Material, further inquiries can be directed to the corresponding author/s.

## Ethics Statement

The studies involving human participants were reviewed and approved by the Ethics Committee of the Affiliated Hospital of Qingdao University. The patients/participants provided their written informed consent to participate in this study. Written informed consent was obtained from the individual(s) for the publication of any potentially identifiable images or data included in this article.

## Author Contributions

CL designed the study with XX and JL. CL and JL had full access to all of the data in the study and took responsibility for the integrity of the data and the accuracy of the data analysis. XX, JL, and TM were major contributors in writing the manuscript. XX, XY, XL, LC, ZL, WS, LH, FY, YH, AJ, and CW have worked on the sample processing. XY, JL, XX, and TM contributed in the interpretation of data. XX, XY, and LH contributed equally to this article. All authors contributed to the article and approved the submitted version.

## Funding

This study was sponsored by the research project grants from National Key Research and Development Program (#2016YFC0903401) and National Science Foundation of China (#81900636, # 82170906, #81602258, #31900413, and #81770869).

## Conflict of Interest

The authors declare that the research was conducted in the absence of any commercial or financial relationships that could be construed as a potential conflict of interest.

## Publisher's Note

All claims expressed in this article are solely those of the authors and do not necessarily represent those of their affiliated organizations, or those of the publisher, the editors and the reviewers. Any product that may be evaluated in this article, or claim that may be made by its manufacturer, is not guaranteed or endorsed by the publisher.

## Abbreviations

ALT: Alanine aminotransferase
AST: Aspartate aminotransferase
Cr: Creatinine
GLU: Serum glucose
TC: Cholesterol
TG: Triglyceride
SU: Serum urate
BUN: blood urea nitrogen
MSU: Monosodium urate
FEUA: Fractional excretion of urate
UUE: Urinary urate excretion
ULN: Upper limit of normal
eGFR: Estimated glomerular filtration rate
BMI: Body mass index.
