# Supplementary figures and images for: Effect of Clinical Typing on Serum Urate Targets of Benzbromarone in Chinese Gout Patients: A Prospective Cohort Study
Source: Front Med (Lausanne). 2022 Jan 17;8:806710. doi: 10.3389/fmed.2021.806710 (PMC8801777; doi:10.3389/fmed.2021.806710)

**Supplementary Figure 1.**

**
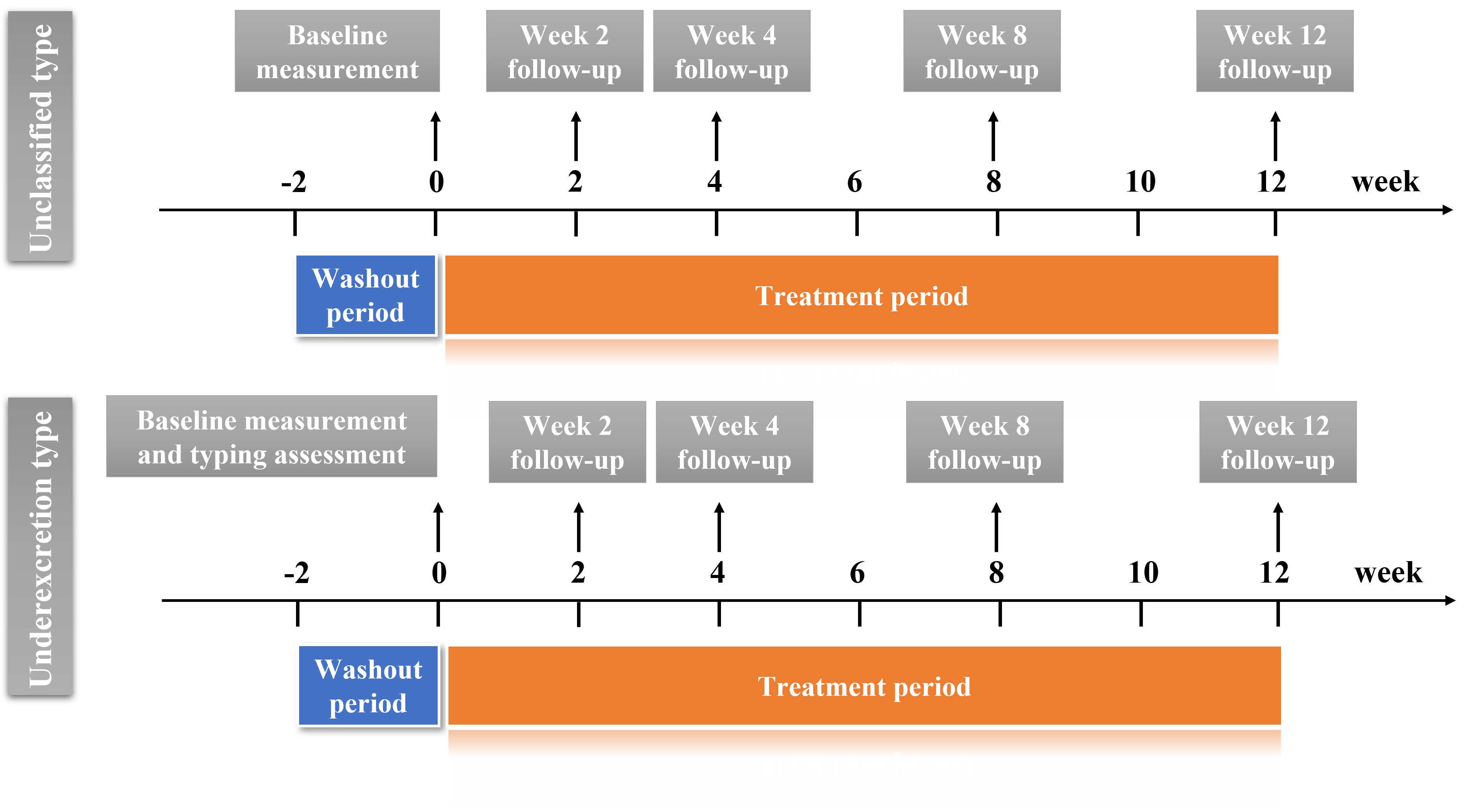
**

Supplement: Supplementary file 1 [file Data_Sheet_1.DOCX]
