# Supplementary material for: Effect of Clinical Typing on Serum Urate Targets of Benzbromarone in Chinese Gout Patients: A Prospective Cohort Study
Source: Front Med (Lausanne). 2022 Jan 17;8:806710. doi: 10.3389/fmed.2021.806710 (PMC8801777; doi:10.3389/fmed.2021.806710)
Supplement: Supplementary file 2 [file Table_1.DOCX]

**Supplementary table 1. Incidence of treatment-emergent adverse events during the study**

|  | **Unclassified Type**  **(n = 109)** | **Underexcretion Type**  **(n = 100)** | ***P* value** |
| --- | --- | --- | --- |
| **Gout flare, n (%)** | 25 (22.9) | 30 (30.0) | 0.25 |
| **once** | 22 (20.2) | 22 (22.0) | 0.75 |
| **twice** | 3 (2.8) | 7 (7.0) | 0.20 |
| **> twice** | 0 (0) | 1 (1) | 0.48 |
| **Any transaminase elevation from normal, n (%)** | 21 (19.3) | 17 (17.0) | 0.67 |
| **1~2 × ULN, n (%)** | 18 (16.5) | 14 (14.0) | 0.61 |
| **2~3 × ULN, n (%)** | 3 (2.8) | 2 (2.0) | 1.00 |
| **>3 × ULN, n (%)** | 0 (0) | 1 (1.0) | 0.48 |
| **Any eGFR < 60 mL/min/1.73m^2^, n (%)** | 0 (0) | 1 (1.0) | 0.48 |
| **Renal calculi, n (%)** | 8 (7.3) | 6 (6.0) | 0.70 |
| **Gastrointestinal disorders, n (%)** | 0 (0) | 3 (3.0) | 0.11 |
| **Cardiovascular events (Increased heart rate), n (%)** | 0 (0) | 2 (2.0) | 0.23 |
| **Skin and subcutaneous tissue disorders (Itchy skin), n (%)** | 0 (0) | 1 (1.0) | 0.48 |
| **Respiratory, thoracic and mediastinal disorders, n (%)** | 0 (0) | 1 (1.0) | 0.48 |
| **Others, n (%)** | 0 (0) | 0 (0) | 1.00 |

Data were presented as percentages. ULN: upper limit of normal; eGFR: estimated glomerular filtration rate.
